# A case of diaphragmatic hemangioma completely resected by partial diaphragmatic resection

**DOI:** 10.1093/jscr/rjac262

**Published:** 2022-06-07

**Authors:** Hiroki Mori, Naohiro Nose, Tomoka Hamahiro, Yoshiya Shimao, Masaki Tomita, Koji Furukawa

**Affiliations:** Department of General Thoracic Surgery, Miyazaki Prefectural Nobeoka Hospital, Miyazaki, Japan; Department of General Thoracic Surgery, Miyazaki Prefectural Nobeoka Hospital, Miyazaki, Japan; Department of General Thoracic Surgery, Miyazaki Prefectural Nobeoka Hospital, Miyazaki, Japan; Department of Pathology, Miyazaki Prefectural Nobeoka Hospital, Miyazaki, Japan; Department of Thoracic and Breast Surgery, Faculty of Medicine University of Miyazaki, Miyazaki, Japan; Department of Cardiovascular Surgery, Faculty of Medicine University of Miyazaki, Miyazaki, Japan

## Abstract

Diaphragmatic hemangiomas are rare tumors and the preferred resection range in surgical procedures is considered on a case-by-case basis. We report a case of diaphragmatic hemangioma that was completely resected by partial diaphragmatic resection. An 81-year-old man was referred for the examination of right diaphragmatic mass. Computed tomography revealed two contrast-enhanced nodules (diameter: 17 and 10 mm, respectively) on the right diaphragm. The nodules were completely resected by partial resection of the diaphragm via video-assisted thoracic surgery using an ultrasonic coagulation and incision device. Resection was performed leaving part of the muscular layer of the diaphragm. Histopathology confirmed the nodule to be hemangioma originating from the diaphragm and no hemangiomatous lesions were noted in the normal connective tissue in the resected stump. Partial diaphragmatic resection is a less invasive treatment method and may be a useful surgical procedure for diaphragmatic hemangioma.

## INTRODUCTION

Primary tumors of the diaphragm are rare and there have been few case reports [1–10] on diaphragmatic hemangioma. Given the rarity of this disease, the appropriate range of surgical resection has not been determined. We herein report a case of diaphragmatic hemangioma that was completely resected by partial resection of the diaphragm via video-assisted thoracic surgery (VATS).

## CASE REPORT

**Figure 1:**
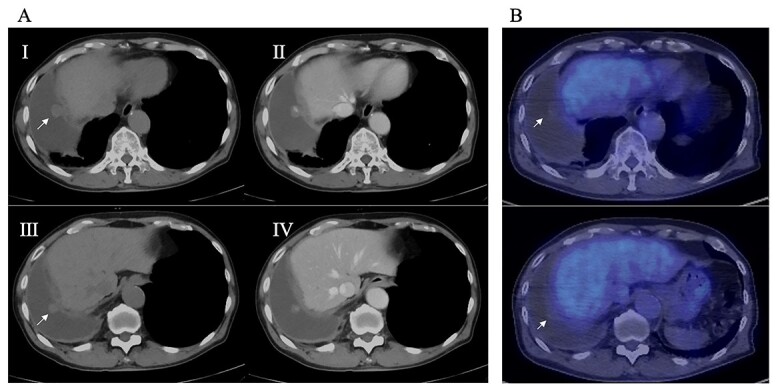
(**A**) Preoperative CT; right pleural effusion was present, and a 17 × 10 mm nodule (I) and a 10 × 10 mm nodule (III) were found on the right diaphragm; contrast medium staining from the center (II, IV), and (**B**) preoperative PET-CT; no accumulation of FDG was observed in the diaphragmatic nodules.

**Figure 2:**
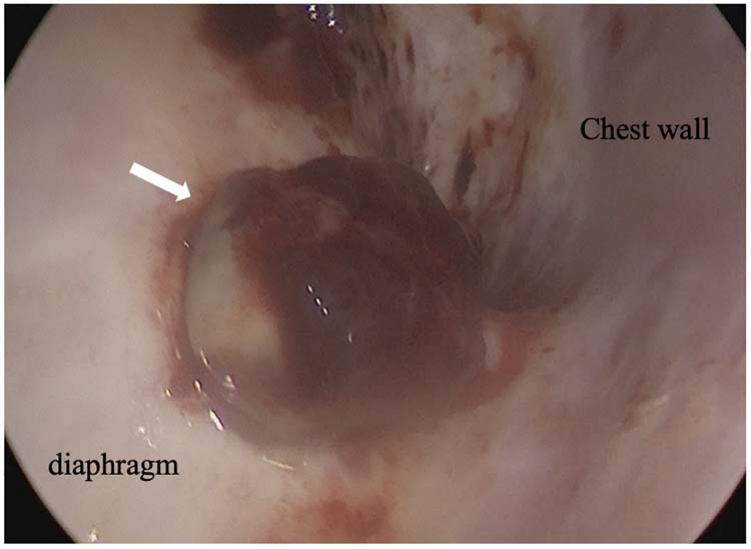
Intraoperative thoracoscopic image, and two consecutive reddish, smooth nodules were observed on the surface of the diaphragm.

**Figure 3:**
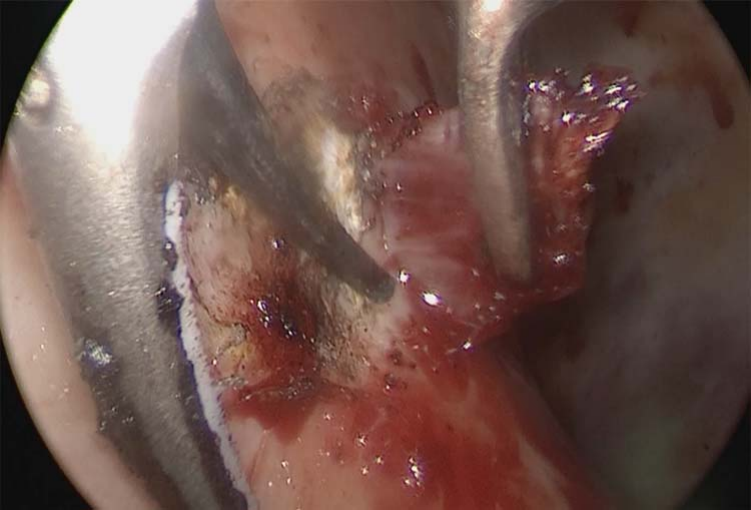
Intraoperative thoracoscopic image, and the diaphragm layer was peeled off with the active blade of the ultrasonic coagulation and incision device.

**Figure 4:**
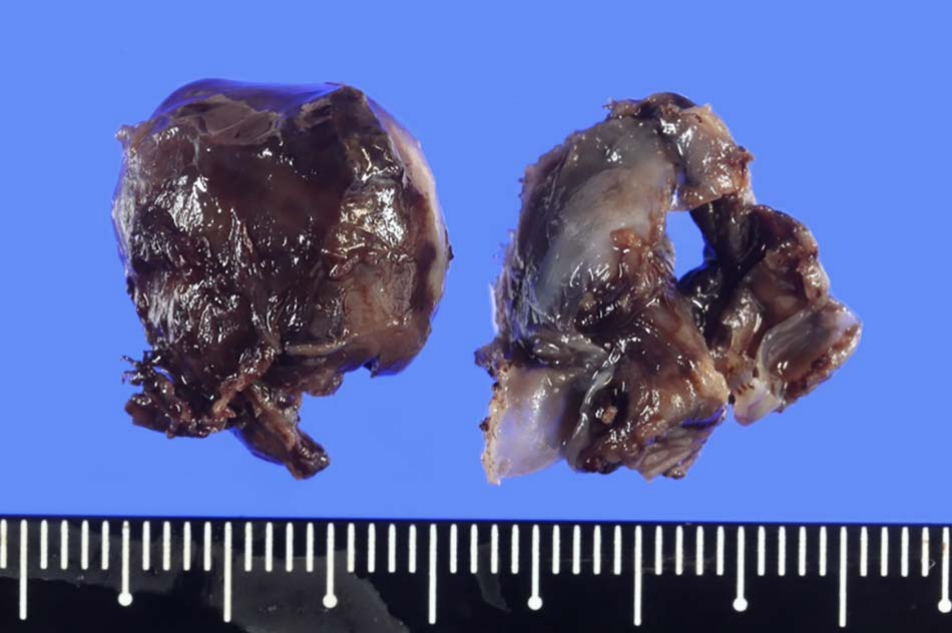
Excised hemangioma, and the nodules were 1.7 × 1.7 cm and 1.5 × 1.5 cm with a brown surface.

**Figure 5:**
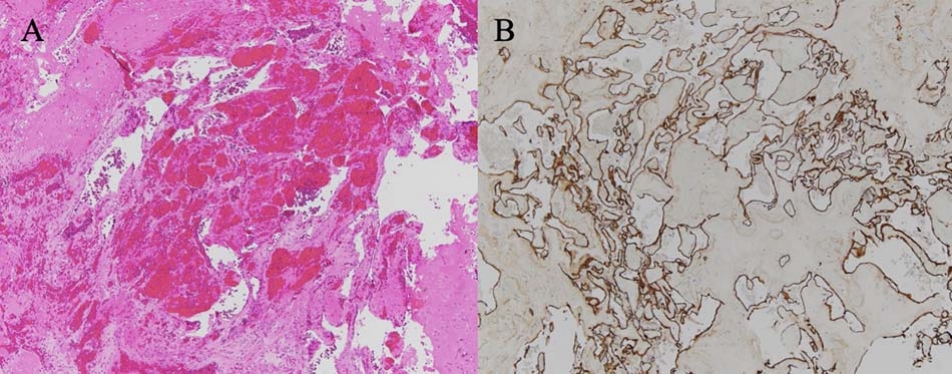
(**A**) HE-stained image of the specimen, and a collection of blood vessels was observed; (**B**) CD34 staining was positive, confirming the presence of vascular endothelium.

**Figure 6:**
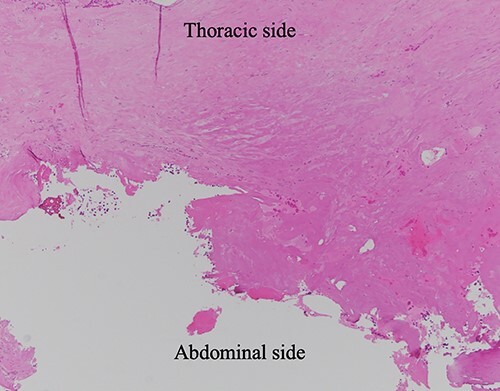
HE-stained image of the specimen with additional diaphragm excision, and the lower side of the photo shows the exfoliated surface of the diaphragm, which turned red due to tissue cauterization; no residual hemangioma structure was found.

An 81-year-old man who underwent surgical resection for lung cancer 2 years previously was referred for the examination of a right diaphragmatic mass that was pointed out during follow-up. Computed tomography (CT) revealed pleural effusion and two contrast-enhanced nodules (17 × 10 and 10 × 10 mm) in the right diaphragm (Fig. 1A); however, he had no symptoms. Positron emission tomography (PET)/CT was performed, but no FDG accumulation was found (Fig. 1B). Although hemangioma was suspected based on the preoperative imaging, we were unable to exclude the recurrence of lung cancer. Therefore, the patient was admitted for the examination of the right diaphragmatic nodules by VATS. A 4-cm thoracotomy wound was created at the 8th intercostal mid-axillary line, and a 5.5-mm port was created at the 6th intercostal anterior axillary line. Observation of the thoracic cavity revealed reddish brown pleural effusion and two large and small pedunculated tumors on the diaphragm (Fig. 2). The smaller nodule spontaneously fell off during the process of cleaning the thoracic cavity and was submitted for a rapid intraoperative diagnosis. As hemangiomas were diagnosed during the operation, the roots of the smaller nodule and the larger nodule that fell off were treated using an ultrasonic coagulation and incision device while partially leaving the abdominal layer of the diaphragm. Partial excision was performed (Fig. 3). The nodules were 1.7 × 1.7 and 1.5 × 1.5 cm with a brown surface (Fig. 4). Histopathologically, the nodules were mostly blood clots, and a collection of thin-walled blood vessels with red blood cells was observed (Fig. 5A). An immunohistochemical analysis revealed that the vascular endothelial cells were positive for CD31 and CD34 (Fig. 5B). Thus, the nodules were diagnosed as diaphragmatic hemangioma. No hemangiomatous lesions were noted in the normal connective tissue in the additional resected tissue specimens (Fig. 6). The postoperative course was normal, and the patient is being followed up in an outpatient setting. It has been 16 months since the operation, but no recurrence has been observed at this time.

**Table 1:** Reviews of eleven cases of diaphragmatic hemangioma in the English literature

No	Year	Author	Age	Sex	Clinical symptoms	Diagnostic modality	Site	Size	Approach	Repair of diaphragm	Recurrence
1	1999	Kaniklides *et al.*	4	M	None	CT, MRI, angiography	Supraphrenic, left	50 × 90 mm	Surgical resection with laparotomy plus thoracotomy	Lyo-dura patch	None
2	2000	Ohsaki *et al.*	31	F	Chest pain on deep inspiration	CT, MRI, bone scintigraphy	Supraphrenic, right	60 × 60 mm	Surgical resection via thoracic approach	Unknown	None
3	2001	Cacciaguerra *et al.*	0	F	Neonatal respiratory failure and hydrops fetalis	Cardiac echography, CT	Supraphrenic, right	40 mm	Surgical resection with median sternotomy	Gore-tex patch, plication	None
4	2006	Kono *et al.*	75	F	None	CT, MRI	Supraphrenic, right	25 mm	Surgical resection via thoracoscopic approach	Unknown	None
5	2010	Ino *et al.*	64	M	None	CT, PET-CT	Subphrenic, left	20 mm	Surgical resection via laparoscopic approach	Unknown	None
6	2011	Tsang *et al.*	0	M	Massive pleural effusion, pericardial effusion with cardiac tamponade	Cardiac echography	Supraphrenic and subphrenic, left	60 × 50 mm	Surgical resection with median sternotomy, extended to upper abdomen	Gore-tex patch	None
7	2013	Ueno *et al.*	51	M	None	CT, PET-CT	Supraphrenic, right	17 × 10 mm	Surgical resection via thoracic approach	Partial resection of the diaphragm with staplers	None
8	2013	Yao *et al.*	0	Unknown	Dyspnea	CT, MRI	Supraphrenic, right	Unknown	Interventional vascular embolization	-	None
9	2015	Wu *et al.*	0	F	Progressive respiratory distress and massive right hydrothorax	Ultrasonography, CT, MRI	Supraphrenic, right	Unknown	Interventional vascular embolization	-	None
10	2019	Chu *et al.*	40	M	None	CT	Supraphrenic, right	31 × 15 mm	Surgical resection via thoracic approach	Primary repair	None
11	2021	Our case	81	M	None	CT, PET	Supraphrenic, right	17 × 10 mm	Surgical resection via thoracic approach	None	None

## DISCUSSION

Diaphragmatic tumors are rare, with less than 200 cases reported in the literature to date since the first report by Grancher in 1868 [11]. Kim reported that 58% of the previously reported 144 diaphragmatic tumors were benign tumors, while the remaining 42% were malignant tumors [11]. Of these benign tumors, only 10 cases of diaphragmatic hemangioma have been reported in the relevant English literature [1–10].

Kim reported that the common symptoms of diaphragmatic tumors are chest pain and abdominal pain and that the proportion of cases in which diaphragmatic tumors were incidentally found was 10–15% [11]. Eleven reported cases, including our own, are shown in Table 1 [1–10]. Six reported cases of diaphragmatic hemangioma were asymptomatic, and 5 cases exhibited clinical symptoms. The preoperative diagnoses were reported using dynamic CT, MRI, PET-CT and bone scintigraphy. Due to the nature of hemangioma, a modality that reflects the blood flow may be useful. However, they cannot be distinguished from vascular rich tumors, a definitive diagnosis cannot be made before surgery. For this reason, resection is necessary for the diagnosis and treatment [2].

Regarding surgery, there are two main resection methods. In previous reports, when the tumor size exceeded 40 mm, full-thickness resection and reconstruction were performed using Gore-Tex sheet. On the other hand, for tumors of 20–30 mm or smaller, partial resection with an automatic suture machine or direct suturing after full-thickness resection has been performed. In our case, malignancy was ruled out during the operation. Therefore, the base of the tumor was also resected, leaving part of the muscular layer of the diaphragm. The diaphragm has multiple layered structures [12] allowing us to resect the tumor while leaving part of the diaphragm. It is difficult to set guidelines regarding the size of tumors that can be adapted; however, considering the reports thus far, a size of 20–30 mm or smaller is a reasonable range. As this layered structure does not exist in the center of the tendon, it is difficult to partially resect the tumor and diaphragm. If it extends to the center of the tendon or if the lesion is large and occupies most of the diaphragm, full-thickness excision and reconstruction of the diaphragm are required.

In partial resection in the clinical setting, the use of an electric scalpel is difficult because electrical stimulation stimulates the movement of the diaphragm. Therefore, in this case, the operation was performed using the active blade of the ultrasonic coagulation and incision device, and the root excision operation was completed.

No residual hemangioma lesion was noted in the normal connective tissue in the pathological specimen after additional resection of the diaphragmatic stump in this case (Fig. 6), thus demonstrating that this surgical procedure (a partial resection) is appropriate for diaphragmatic hemangioma.

No recurrence has been reported in the surgical cases of diaphragmatic hemangioma to date; however, recurrence has been reported in previous reports on systemic hemangioma [13]; thus, we were careful in determining the resection range. On the other hand, it is beneficial to avoid surgical invasion as much as possible for benign tumors such as hemangiomas. In this case, 16 months have passed since the operation, but no recurrence has been observed. Although the number of cases is small and more cases are needed to determine the optimal surgical procedure, partial excision that leaves a layer may be a useful surgical procedure.
